# Evidence of a dissociation pattern in default mode subnetwork functional connectivity in schizophrenia

**DOI:** 10.1038/srep14655

**Published:** 2015-09-30

**Authors:** Huaning Wang, Ling-Li Zeng, Yunchun Chen, Hong Yin, Qingrong Tan, Dewen Hu

**Affiliations:** 1 Department of Psychiatry, Xijing Hospital of the Fourth Military Medical University, Xi’an Shaanxi 710032, People’s Republic of China; 2 College of Mechatronics and Automation, National University of Defense Technology, Changsha, Hunan 410073, People’s Republic of China; 3 Department of Radiology, Xijing Hospital of the Fourth Military Medical University, Xi’an Shaanxi 710032, People’s Republic of China

## Abstract

The default mode network (DMN) is suggested to play a pivotal role in schizophrenia; however, the dissociation pattern of functional connectivity of DMN subsystems remains uncharacterized in this disease. In this study, resting-state fMRI data were acquired from 55 schizophrenic patients and 53 matched healthy controls. DMN connectivity was estimated from time courses of independent components. The lateral DMN exhibited decreased connectivity with the unimodal sensorimotor cortex but increased connectivity with the heteromodal association areas in schizophrenics. The increased connectivity between the lateral DMN and right control network was significantly correlated with negative and anergia factor scores in the schizophrenic patients. The anterior and posterior DMNs exhibited increased and decreased connectivity with the right control and lateral visual networks, respectively, in schizophrenics. The altered DMN connectivity may underlie the hallucinations, delusions, thought disturbances, and negative symptoms involved in schizophrenia. Furthermore, DMN connectivity patterns could be used to differentiate patients from controls with 76.9% accuracy. These findings may shed new light on the distinct role of DMN subsystems in schizophrenia, thereby furthering our understanding of the pathophysiology of schizophrenia. Elucidating key disease-related DMN subsystems is critical for identifying treatment targets and aiding in the clinical diagnosis and development of treatment strategies.

Schizophrenia is a psychotic disorder that impairs multiple cognitive domains, including perception, memory, attention, and executive function, as evidenced by delusions, hallucinations, disorganized speech and thought formation, social withdrawal, gross disorganization, and other negative symptoms^1^. To date, the causes and mechanisms of schizophrenia remain unclear. However, it has been proposed that the pathophysiology of schizophrenia is associated with the dysfunctional integration of distributed neuronal networks rather than with the breakdown in the function of a single discrete brain region^2^,^3^.

Recently, the default mode network (DMN) has attracted increasing attention in the study of psychiatric disorders^4^, including schizophrenia^5^,^6^,^7^,^8^. The DMN, which is primarily composed of the medial prefrontal cortex, posterior cingulate cortex/precuneus, bilateral inferior parietal lobule, and temporal cortex^9^,^10^, has been suggested to subserve internal mentation^11^, including autobiographical memory, future planning, theory of mind, self-reference, and affective decision making^12^,^13^. Based on resting-state functional magnetic resonance imaging (fMRI), Mingoia *et al.* reported increased functional connectivity within the DMN in schizophrenics^14^. Using working memory-related paradigms, several task-related fMRI studies demonstrated that deficits in working memory in schizophrenics may be related to the abnormal activity and connectivity of the DMN^7^,^15^,^16^. Furthermore, investigation of schizophrenics before and after olanzapine treatment demonstrated that this treatment may be associated with the modulation of DMN connectivity^17^ and that functional abnormalities in the DMN were correlated with structural changes in schizophrenic patients^18^. In addition to the intra-default connectivity (i.e., within the DMN), inter-default functional connectivity abnormalities were observed between the DMN and ventral attention and frontoparietal control networks in schizophrenic patients relative to healthy controls^19^,^20^, suggesting a central role for the DMN in schizophrenia.

The DMN is not as homogenous as previously assumed; it can be divided into several distinct functional subcomponents (or subnetworks) in the human brain^21^,^22^,^23^,^24^. Previous work supports the hypothesis that anterior DMN is mainly involved in self-referential processing, while the posterior DMN is involved in familiarity and/or autobiographical memory^25^,^26^. In particular, several resting-state fMRI studies have demonstrated a dissociation in DMN functional connectivity in psychiatric and neurologic disorders, including depression^27^ and Alzheimer’s disease^28^. Damoiseaux and colleagues showed decreased connectivity in the posterior DMN but increased connectivity in the anterior and ventral DMN in patients with Alzheimer’s disease^28^. Li *et al.* demonstrated that changes in functional connectivity in the posterior DMN could be normalized with antidepressant treatment, while abnormal functional connectivity persisted within the anterior DMN in depression^27^. The DMN has been extensively investigated in schizophrenia, but it is unknown whether there is a dissociation pattern within the DMN subnetwork that is correlated with the disorder. In particular, the dissociation of functional connectivity between DMN subsystems and all other intrinsic connectivity networks (ICNs) in schizophrenia remains uncharacterized. Repetitive transcranial magnetic stimulation, as a non-invasive brain stimulation technique, is useful for treating a variety of neuropsychiatric disorders (including schizophrenia^29^) but lacks target specificity. Separating key disease-related default mode subsystem from other areas in schizophrenics may provide useful information for the identification of treatment targets and the development of treatment strategies, while shedding light on the pathophysiology of the disorder.

In the present study, we used group independent component analysis (ICA) with default parameter settings to extract the ICNs (referred to as “temporal coherent networks” in group ICA) and corresponding time courses from resting-state fMRI data collected from 55 schizophrenic patients and 53 matched healthy controls^6^. Through this analysis, we identified three DMN subcomponents (anterior, posterior, and lateral ones) and 11 other ICN components relevant in schizophrenia. We then explored intra-default connectivity by estimating the temporal correlation between the ICA component time courses corresponding to DMN subcomponents and the inter-default connectivity by estimating the temporal correlation between DMN subcomponent time courses and the time courses of all other ICNs (i.e., components). Next, group differences in intra- and inter-default connectivity between schizophrenic patients and healthy controls were identified by statistical analysis. In addition, we grouped the ICNs into unimodal sensorimotor cortical networks supporting low-level unimodal processing (i.e., the somatomotor, motor, auditory, and visual cortices) and heteromodal association cortical networks supporting high-level cognitive processing (i.e., the default mode, precuneus, control, and attention networks)^24^ and performed group-level comparison. Finally, we used multivariate pattern analysis methods combining the linear support vector machine (SVM) with local linear embedding^30^ to differentiate schizophrenic patients from healthy controls based on DMN functional network connectivity, thus lending support to the hypothesis that there is a dissociation pattern in functional connectivity between DMN subsystems and other ICNs in schizophrenia.

## Results

### Identification of ICNs

**Figure 1:**
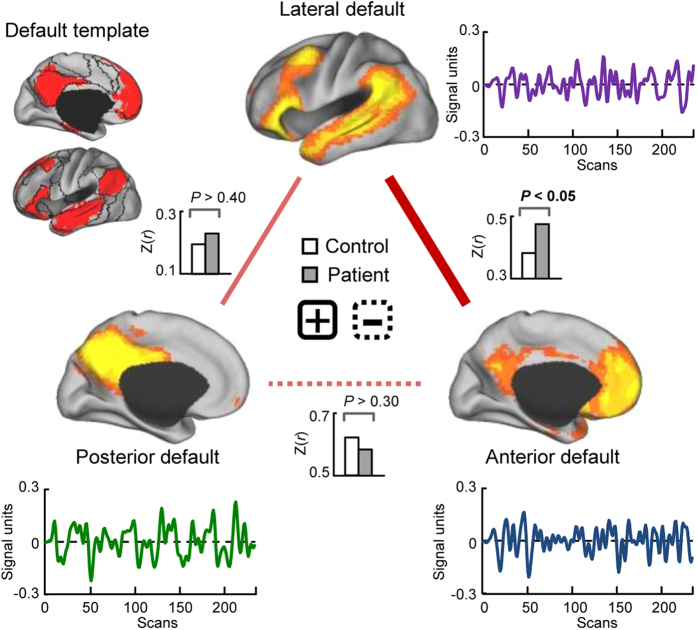
Comparison of intra-default functional connectivity between schizophrenic patients and healthy controls (two-sample *t*-test, *P* < 0.05, uncorrected). The upper left panel presents the default mode network (DMN) template based on prior parcellation of the cerebrum^22^. The spatial expression and corresponding time courses of the DMN subnetworks for the two groups are also presented (one-sample *t*-test, *P* < 0.05, FWE corrected). The anterior DMN mainly consists of the medial prefrontal cortex and portions of the posterior cingulate cortex and bilateral parietal cortex. The posterior DMN consists predominantly of the bilateral precuneus and posterior cingulate cortex, as well as part of the bilateral parietal cortex. The lateral DMN primarily consists of the bilateral parietal cortex, bilateral temporal cortex, and part of the lateral prefrontal cortex. It was observed that positive functional connectivity between the lateral and anterior DMNs was increased in schizophrenic patients relative to healthy controls (*P* < 0.05, uncorrected), and in non-medicated patients (n = 34) relative to healthy controls (*P* < 0.05, uncorrected). The red lines represent positive functional connectivity. The solid and dashed lines represent an increase and decrease in schizophrenic patients relative to healthy controls, respectively. CARET software (CARET; http://brainvis.wustl.edu) was used for surface rendering.

Fourteen functionally relevant ICNs were identified by group ICA analysis according to the previous studies^23^,^24^, as shown in Supplementary Fig. 1 (one-sample *t*-test, *P* < 0.05, FWE corrected). Three components, or subnetworks, of the DMN were identified: an anterior subnetwork with a correlation (over voxels) of 0.36 (*P* < 0.001) with the DMN template; a posterior subnetwork with a correlation of 0.19 (*P* < 0.001); and a lateral subnetwork with a correlation of 0.38 (*P* < 0.001) (Supplementary Fig. 1). The corresponding time courses of the three subnetworks are shown in Fig. 1. The anterior DMN had the highest amplitude in the medial prefrontal cortex, the posterior DMN had the highest amplitude in the posterior cingulate cortex/precuneus, and the lateral DMN had the highest amplitude in the bilateral temporal cortex. The three networks are spatially independent and their time courses are asynchronous.

### Intra-default functional connectivity

The intra-default functional connectivity was calculated for each subject, and the correlation value between each pair of the DMN subnetwork was positive and significant for both control and patient groups (Supplementary Fig. 2; one-sample *t*-test, *P* < 0.05, FDR corrected). Two-sample *t*-tests revealed that the functional connectivity between the lateral and anterior DMNs was significantly increased in schizophrenic patients relative to healthy controls (Fig. 1, *P* < 0.05, uncorrected) as well as in non-medicated patients (n = 34) relative to healthy controls (*P* < 0.05, uncorrected). No significant difference was observed in intra-default functional connectivity between non-medicated and medicated patients.

### Inter-default functional connectivity

**Figure 2:**
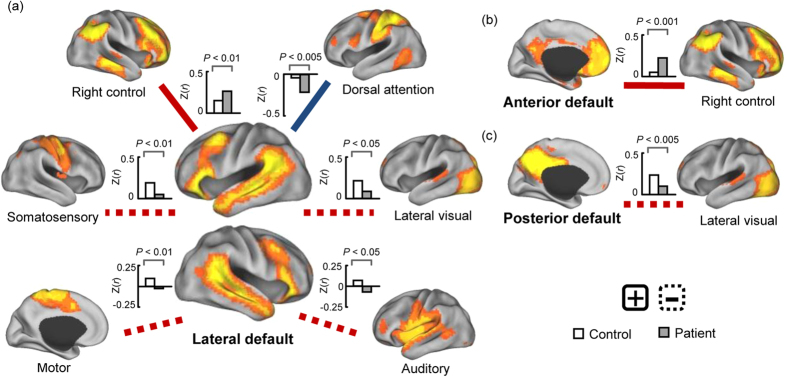
Functional connectivity was significantly altered between the default mode subnetworks and other intrinsic connectivity networks in schizophrenic patients relative to healthy controls (two-sample *t*-test, *P* < 0.05, FDR corrected). The connections, as shown, were also significantly altered in non-medicated patients (n = 34) relative to healthy controls (two-sample *t*-test, *P* < 0.05, uncorrected). The red and blue lines represent positive and negative functional connectivity, respectively. The solid and dashed lines represent an increase and decrease in schizophrenic patients relative to healthy controls, respectively. CARET software (CARET; http://brainvis.wustl.edu) was used for surface rendering.

Taking each DMN subnetwork as a seed network, the inter-default functional connectivity was calculated between seed networks and other ICNs for each subject. The results of one-sample *t*-tests for each group can be observed in Supplementary Figs 3–5 (*P* < 0.05, FDR corrected). Two-sample *t*-tests revealed that the functional connectivity between the lateral DMN and right frontoparietal control (positive) and dorsal attention networks (negative) was significantly enhanced in schizophrenic patients relative to healthy controls (Fig. 2a, *P* < 0.05, FDR corrected), while the functional connectivity (positive) between the lateral DMN and somatomotor, motor, lateral visual and auditory cortices was significantly reduced in schizophrenic patients (Fig. 2a, *P* < 0.05, FDR corrected). Meanwhile, the functional connectivity between the anterior DMN and right frontoparietal control network (positive) was significantly increased in schizophrenic patients (Fig. 2b, *P* < 0.05, FDR corrected), while the functional connectivity between the posterior DMN and lateral visual cortex (positive) was significantly decreased in schizophrenic patients (Fig. 2c, *P* < 0.05, FDR corrected). All connections, as shown in Fig. 2, were significantly altered in non-medicated patients (n = 34) relative to healthy controls (*P* < 0.05, uncorrected). In a comparison between medicated and non-medicated patients, two negative connections were significantly increased in medicated patients relative to non-medicated patients (*P* < 0.05, FDR corrected): those between the anterior DMN and precuneus and between the anterior DMN and dorsal attention network.

**Figure 3:**
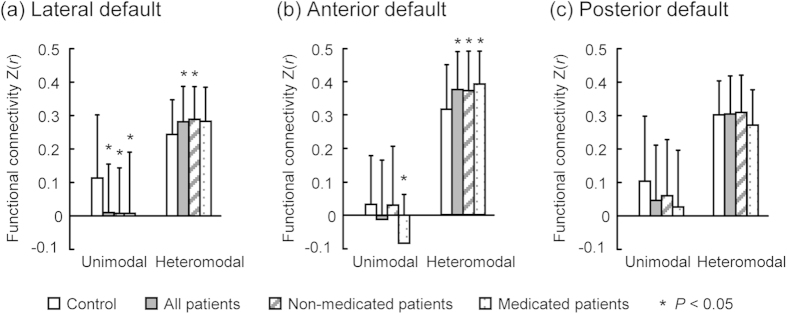
The dissociation pattern of the inter-default functional connectivity within unimodal and heteromodal cortical networks (two-sample *t*-test, *P* < 0.05, uncorrected). Note that functional connectivity was significantly decreased between the lateral default mode network (DMN) and unimodal cortical networks in both non-medicated and medicated patients, while it was increased between the lateral DMN and heteromodal cortical networks in the non-medicated patients only (**a**). The functional connectivity between the anterior DMN and heteromodal cortical networks was significantly increased in both non-medicated and medicated patients (**b**). No significant difference in functional connectivity was detected between the posterior DMN and unimodal and heteromodal cortical networks (**c**), and no significant difference in the aforementioned functional connectivity was observed between the medicated and non-medicated patients.

When the 14 ICNs were grouped into unimodal sensorimotor cortical networks (i.e., somatomotor, motor, auditory, and visual cortices) and heteromodal association cortical networks (i.e., default mode, precuneus, control, and attention networks)^24^, we observed that functional connectivity was significantly decreased between the lateral DMN and unimodal cortical networks in both non-medicated and medicated patients, while functional connectivity was significantly increased between lateral DMN and heteromodal cortical networks in non-medicated patients only (Fig. 3a, *P* < 0.05, uncorrected). In addition, functional connectivity between the anterior DMN and heteromodal cortical networks was significantly increased in both non-medicated and medicated patients (Fig. 3b, *P* < 0.05, uncorrected). No significant functional connectivity difference was detected between the posterior DMN and unimodal and heteromodal cortical networks (Fig. 3c, *P* < 0.05, uncorrected), and no significant difference in functional connectivity was observed between medicated and non-medicated patients (*P* < 0.05, uncorrected).

### Clinical correlation analysis

**Figure 4:**
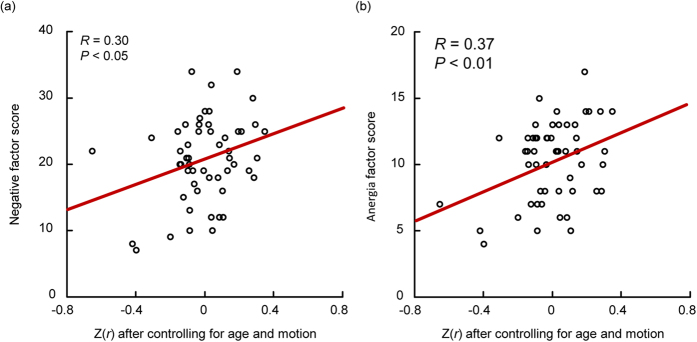
The functional connectivity between the lateral default mode and right frontoparietal control networks is significantly correlated with PANSS negative factor scores (a, *R* = 0.30, *P* < 0.05) and anergia factor scores (b, R = 0.37, P < 0.01) in schizophrenic patients.

The functional connectivity between lateral DMN and right frontoparietal control network was positively correlated with Positive and Negative Syndrome Scale (PANSS) negative factor scores (*R* = 0.30, *P* < 0.05, uncorrected) and anergia factor scores (*R* = 0.37, *P* < 0.01, uncorrected), after controlling for age and head motion (Fig. 4) and when thepoints corresponding to the patient with largest PANSS total score (208≫ mean score) and lowest PANSS positive factor score (7≪ mean score) were treated as ‘outliers’ and were removed.

### Classification results

Classification results demonstrated that anterior, posterior, and lateral DMN functional network connectivity, respectively, could be used to differentiate schizophrenic patients from healthy controls with peak accuracies of 71.3% (69.1% for patients and 73.6% for controls; *P* < 0.0001), 63.9% (61.8% for patients and 66.0% for controls; *P* < 0.005), and 73.2% (74.6% for patients and 71.7% for controls; *P* < 0.0001) via leave-one-out cross-validation; the corresponding averaged classification rates of the training datasets were 78.6%, 67.3%, and 77.0%, respectively. When the functional network connectivity of all the three subnetworks was taken together as classification features, an accuracy of 76.9% (80.0% for patients and 73.6% for controls; *P* < 0.0001) was achieved, and the corresponding averaged classification rates of the training datasets were 78.6%.

## Discussion

In this study, we investigated the functional network connectivity of DMN subnetworks in schizophrenic patients using group ICA of resting-state fMRI scans. Three components, or subnetworks, associated with the DMN were identified: the anterior, posterior and lateral DMNs. We observed that the intra-default functional connectivity between the anterior and lateral DMNs was significantly increased in schizophrenic patients relative to healthy controls. The calculation of inter-default functional correlation revealed that the lateral DMN exhibited decreased functional connectivity with unimodal cortical networks and increased functional connectivity with heteromodal cortical networks in the patient group. The increased functional connectivity between the lateral DMN and right control network was significantly correlated with PANSS negative and anergia factor scores in schizophrenic patients. Furthermore, the functional connectivity between the anterior DMN and right control network and between the posterior DMN and lateral visual network were significantly increased and decreased in schizophrenics, respectively. In addition, DMN functional network connectivity could be used to differentiate patients from controls with an accuracy of 76.9%. These findings provide new insight into the distinct roles of DMN subsystems in schizophrenia, thereby developing our understanding of the pathophysiology of schizophrenia. Differentiating key disease-related DMN subsystems from those not involved is critical for identifying treatment targets and the developing treatment strategies for schizophrenia.

The DMN has been suggested to underlie external environment monitoring, spontaneous cognition, and autobiographical thinking^12^,^13^. General symptoms of schizophrenia include delusion, autistic thinking, and the splitting of autobiographical thought, therefore suggesting that dysfunction of the DMN may play a role in the disorder. In the current study, the DMN was separated into the lateral, anterior, and posterior subsystems, which exhibit distinct, abnormal inter-default functional network connectivity patterns in schizophrenic patients relative to healthy controls. Among the three subsystems, the functional connectivity between the anterior and lateral DMN was enhanced in schizophrenic patients, consistent with the majority of previous studies^7^,^14^. Similar results were obtained for the non-medicated patient group alone. As the anterior hub of the DMN, the medial prefrontal cortex is critical in self-referential processing. Abnormalities in this region have been suggested to underlie the impairment in reality monitoring characteristic of schizophrenia^31^,^32^. During rest, increased activity in the medial prefrontal cortex is correlated with the severity of positive symptoms in schizophrenia^6^; while performing passive tasks, this increase was negatively correlated with cognitive test scores in schizophrenic patients^33^. Furthermore, Whitfield-Gabrieli *et al.* observed that schizophrenic patients exhibited hyperactivity in the medial prefrontal cortex during a working memory task and increased connectivity between the medial prefrontal cortex and other DMN regions, both during rest and a task^7^. A failure in the deactivation of the anterior DMN has also been reported by Pomarol-Clotet and colleagues^15^. Accordingly, our observations may confirm the previous findings, and altered intra-default functional connectivity may contribute to disturbances of thought as well as self- and source-monitoring in schizophrenia.

In this study, the lateral DMN exhibited significantly decreased functional connectivity with unimodal systems, including the somatomotor, motor, lateral visual and auditory networks, in schizophrenic patients, supporting the hypothesis that functional disconnectivity underlies schizophrenia. Similar results were obtained for the non-medicated patients alone. The unimodal sensorimotor cortex is involved in creating representations that convey the visual, auditory and somatomotor information necessary for real-world perception. Note that the lateral DMN overlaps the language network, mainly the bilateral superior medial and inferior frontal cortex (including Broca’s area), the lateral temporal cortex (including Wernicke’s area and the superior temporal sulcus), and the temporal parietal junction. The previous voxel-based morphometry studies reported that gray matter volume reduction in the superior temporal gyrus may be related to the positive symptoms, such as hallucinations and thought disturbances, in schizophrenia^34^. Functional anomalies of the language network were also demonstrated in schizophrenia^35^. Reduced coupling between the lateral DMN and unimodal sensorimotor cortex was not found to be significantly correlated with positive symptoms in the current study; thus, further studies are needed to confirm whether the abnormal modulation of unimodal sensorimotor cortex by the lateral DN underlies the somatomotor, visual, auditory-verbal and multimodality hallucinations characteristic of schizophrenia^33^ that create a vague boundary between internal and external real-world perception.

Previous studies have demonstrated dynamic cooperation and competition between the DMN and other ICNs in the human brain^36^,^37^, perhaps underlying the shift in focus from internal state to external environment. In the current study, the lateral DMN exhibited increased functional connectivity with the right frontoparietal control and dorsal attention networks in schizophrenia, and functional connectivity between the anterior DMN and right control network was enhanced in schizophrenic patients, consistent with the results of previous studies^20^. Similar results were obtained for non-medicated patients alone. Furthermore, the increased functional connectivity between the lateral DMN and right control network was significantly correlated with PANSS negative and anergia factor scores in schizophrenic patients. The frontoparietal control network is suggested to play a central role in cognitive control and information integration from the default mode and dorsal attention systems^38^,^39^. The abnormal coupling between the DMN and control/attention networks implies that schizophrenic brains do not successfully balance the shift of activation and deactivation between these networks (especially for anti-correlated networks). Thus, these anomalies in network coordination may lead to an inability to cycle out of internal thought and into response to external stimuli, resulting in hallucinations, emotional disturbance, social and attention deficits, disrupted corollary and judgment (such as persecutory delusions), and a split between internal mentation and external environment.

We also tested the discriminative power of the functional network connectivity among the anterior, posterior, and lateral DMNs between the patient and control groups, yielding accuracies of 71.3% (*P* < 0.0001), 63.9% (*P* < 0.005), and 73.2% (*P* < 0.0001), respectively. When taking all the three subnetworks together, an accuracy of 76.9% (*P* < 0.0001) was achieved. According to the classification results, the two populations are likely to exhibit DMN connectivity patterns^40^. Recently, several fMRI studies have attempted to distinguish schizophrenic patients from healthy controls^41^,^42^. Our findings suggest that DMN connectivity may also be a potential biomarker for the clinical diagnosis of schizophrenia.

The current study posed several limitations. First, group ICA does not always divide the DMN into three components for all datasets or populations under default parameter settings, but three components can be achieved with a larger component number parameter. Nevertheless, the current study provides preliminary evidence for the functional connectivity dissociation of DMN subnetworks in schizophrenia. Second, the increased functional connectivity between the lateral and anterior DMNs as well as the patterns of functional connectivity between unimodal and heteromodal cortical networks were uncorrected, so these results should be interpreted with caution. Third, IQ was not assessed in the current study, and it remains unclear whether IQ was matched between the patient and control groups. This potential confounding factor should be considered in future studies. Fourth, we estimated static functional connectivity alterations in schizophrenia in this study; dynamic functional connectivity and effective connectivity should be investigated to shed light on the abnormal functional interactions between DMN subnetworks in schizophrenia. Fifth, Salvador *et al.* demonstrated cortical-subcortical functional connectivity abnormalities in schizophrenia^43^. However, due to noise in our data, subcortical systems were not included in the current analyses, and functional connectivity between DMN subsystems and subcortical areas should be examined in the future. Though it remains unclear how these results could be used in clinical practice, differentiating the key disease-related default mode subsystem from others may prove useful in the identification of treatment targets and the development of treatment strategies for schizophrenia, although the DMN subnetwork may not, in practice, be a suitable treatment target in schizophrenia. Finally, due to inter-subject brain differences and scanner variability, it is important to confirm the results with a larger sample size and multicenter imaging data. An expansion of the medicated sample group is ongoing. It is also important to study the influence of antipsychotic medication in schizophrenia in future work.

## Methods

### Participants

Fifty-five patients diagnosed with schizophrenia and fifty-five healthy controls were involved in the study, all of whom were right-handed Chinese speakers. All patients were recruited from the Department of Psychiatry, Xijing Hospital of the Fourth Military Medical University, Xi’an, China. All patients were evaluated with the Structured Clinical Interview for DSM-IV Axis I Disorders (SCID-I) and the Patient Edition (SCID-I/P)^44^ and were required to meet the DSM-IV^1^ diagnostic criteria for schizophrenia. The diagnosis of schizophrenia was confirmed after at least six months. No patients had a history of neurological disorders, severe medical disorders, substance abuse, or electroconvulsive therapy. First-episode patients were included in the current study and 34 of the patients were drug-naïve, while the remainder were receiving antipsychotic medications at the time of image acquisition [risperidone (n = 10, 2–6 mg/day), clozapine (n = 5, 200–350 mg/day), quetiapine (n = 4, 400–600 mg/day), and sulpiride (n = 2, 200 mg/day)]. Patients were considered to be antipsychotic drug-naïve if the following criteria were met: 1) the patient was not receiving antipsychotics at the time of the study; 2) the patient had no history of receiving antipsychotics, based on information provided by two or more family members or care providers; and 3) the patient had no history of antipsychotic prescriptions, based on information provision forms from medical institutions that the patient had visited or a history of prescriptions from pharmacies. The ages of the non-medicated patients were 27.38 ± 5.77 years, and the duration of illness in the non-medicated patients was 11.74 ± 15.04 months, similar to those reported in previous studies^45^. The healthy controls were recruited via local recruitment advertisements in the hospital and in the communities neighboring. All participants provided written informed consent, and the study was approved by the Ethics Committee of the Xijing Hospital of the Fourth Military Medical University. The methods were carried out in accordance to the approved guidelines.

Two healthy controls were discarded due to excessive head motion during scanning acquisition (>2.5 mm translation and/or >2.5° rotation), resulting in the inclusion of 55 schizophrenic patients and 53 healthy controls in follow-up analyses. No significant difference in mean motion was observed between the two groups (patient: 0.066 ± 0.038, control: 0.055 ± 0.024 mm, *P *> 0.05)^46^,^47^. The information collected from the remaining subjects is presented in Table 1, including their sex, age, PANSS, and other factor scores^48^.

### Image acquisition and preprocessing

All data were collected on a 3T Tim Trio scanner (Siemens, Erlangen, Germany) using a 12-channel phased-array head coil. Images were acquired using a gradient-echo echo-planar pulse sequence sensitive to blood oxygenation level-dependent (BOLD) contrast [repetition time (TR)/echo time (TE) = 2000/30 ms; flip angle (FA) = 90°; matrix = 64 × 64; field of view (FOV) = 220; thickness/gap = 4/0.6 mm; slices = 33]. Each resting-state fMRI run lasted 8 min, and each subject underwent two runs. Structural data made use of a high-resolution multi-echo T1-weighted magnetization-prepared gradient-echo image [TR = 2530 ms; Time Inversion (TI) = 1100 ms; TE = 3.5 ms; FA = 7°; 1-mm isotropic voxels; FOV = 256]. Subjects were instructed to stay awake, keep their eyes closed, and minimize head movement; no other task instruction was provided. After each session, the subjects were asked whether they were awake and relaxed in the previous session, and all of the subjects confirmed that they were.

There were no artifacts or structural abnormalities in any of the structural MRI images, as determined by visual inspection. Data were preprocessed using a statistical parametric mapping software package (SPM8, Welcome Department of Cognitive Neurology, Institute of Neurology, London, UK, http://www.fil.ion.ucl.ac.uk/spm). The first five volumes of each run were discarded to allow for T1-equilibration effects. The following steps were performed: 1) slice timing correction; 2) rigid body correction for head motion; 3) atlas registration with an EPI template in the Montreal Neurological Institute (MNI) atlas space, resampling to 3-mm isotropic voxels and spatially smoothing using a 6-mm full-width half-maximum (FWHM) Gaussian kernel; 4) normalization for global mean signal intensity across runs; and 5) linear detrend and band-pass temporal filtering (0.01–0.08 Hz).

### Group independent component analysis

All preprocessed fMRI data were analyzed using GIFT software (http://mialab.mrn.org/software/gift/). Group ICA was performed to decompose the resting-state images into spatially independent components^49^,^50^. For each subject, 20 spatially independent components were obtained with default parameters. The ICNs (14; the remainder were noise components) were identified from the 20 independent components, as reported in previous studies^22^,^23^,^24^. In particular, DMN components (3) were identified using a DMN template based on the prior parcellation of the human brain^22^,^51^,^52^. Operationally, a multiple regression was performed over voxels, and components (i.e., subnetworks) that best fit the DMN template were selected.

### Intra- and inter-default functional connectivity analysis

Taking each DMN subnetwork as a seed network, the intra-default functional correlations between the seed network and other DMN subnetworks as well as inter-default functional correlations between the seed network and other ICNs were estimated from the corresponding component time courses by Pearson correlation coefficient, then were converted to *z*-scores by applying the Fisher *r*-to-*z* transformation^53^. Two-tailed one-sample *t*-tests were used to test whether the functional connectivity between each pair of ICNs was significant for each group (*P* < 0.05, FDR corrected). Finally, two-tailed two-sample *t*-tests were used to compare the intra- and inter-default functional connectivity between the patient and control groups with a threshold of *P* < 0.05 (FDR corrected).

### Clinical correlation analysis

Spearman’s rank correlation analysis was performed to assess the correlation between DMN connectivity with significant group differences and clinical variables, as shown in Table 1. Age and mean motion were included as confounding covariates^47^. Two-tailed two-sample significance levels were set at *P* < 0.05 and were uncorrected for multiple comparisons in the correlation analysis.

### Multivariate pattern analysis

Linear SVMs (http://www.isis.ecs.soton.ac.uk/resources/svminfo/), together with local linear embedding, were used to test the discriminative power of DMN connectivity between the patient and control groups^42^. The algorithms were reported with the best parameter settings. Due to the limited sample size of our study, the leave-one-out cross-validation strategy was used to estimate the generalization ability of the classification. Permutation tests were implemented to assess the performance of the classifier^40^, and the tests were repeated 10,000 times as described previously^54^.

## Additional Information

**How to cite this article**: Wang, H. *et al.* Evidence of a dissociation pattern in default mode subnetwork functional connectivity in schizophrenia. *Sci. Rep.* **5**, 14655; doi: 10.1038/srep14655 (2015).

## Supplementary Material

Supplementary Information

## Figures and Tables

**Table 1:** Demographic and clinical profiles of the participants in this study.

Variable	Schizophrenic patient (mean ± s.d.)	Healthy control (mean ± s.d.)	*p*-value
Sample size	55	53	
Gender (female/male)	23/32	25/28	>0.50^a^
Age (year)	27.27 ± 5.51	28.08 ± 4.68	>0.40^b^
Duration (month)	30.15 ± 34.66		
PANSS total	91.76 ± 21.95		
PANSS positive	22.34 ± 4.49		
PANSS negative	21.07 ± 6.57		
PANSS general	44.45 ± 7.85		
Anergia	10.64 ± 3.43		
Thought disturbance	11.58 ± 3.45		
Activation	6.35 ± 2.35		
Paranoid/Belligerence	9.25 ± 3.20		
Depression	9.78 ± 3.20		
Supplementary items (Attack)	9.55 ± 5.04		

^a^Pearson Chi-square test.

^b^Two-tailed two-sample *t*-test; PANSS = Positive and Negative Syndrome Scale. The following clinical variables were evaluated according to PANSS factors: a) “Anergia” = N1+N2+G7+G10; b) “Thought disturbance” = P2+P3+P5+G9; c) “Activation” = P4+G4+G5; d) “Paranoid/belligerence” = P6+P7+G8; and e) “Supplementary” = P4+P7+G6+S1+S2+S3. P2, Conceptual disorganization; P3, Hallucinations; P4, Hyperactivity; P5, Grandiosity; P6, Suspiciousness/persecution; P7, Hostility; N1, Blunted affect; N2, Emotional Withdrawal; G4, Tension; G5, Mannerisms and posturing; G6, Depression; G7, Motor retardation; G8, Uncooperativeness; G9, Unusual thought content; G10, Disorientation; S1, Angry; S2, Inability to delay gratification; S3, Affective lability.
