# Supplementary material for: Evidence of a dissociation pattern in default mode subnetwork functional connectivity in schizophrenia
Source: Sci Rep. 2015 Sep 30;5:14655. doi: 10.1038/srep14655 (PMC4588504; doi:10.1038/srep14655)
Supplement: Supplementary Information [file srep14655-s1.pdf]

Supporting information

Evidence of a dissociation pattern in default mode subnetwork functional connectivity in schizophrenia

Hua-Ning Wang[1,†], Ling-Li Zeng[2,†], Yunchun Chen[1], Hong Yin[3], Qing-Rong Tan[1,*], Dewen Hu[2,*]

[1] *Department of Psychiatry, Xijing Hospital of the Fourth Military Medical University, Xi'an Shaanxi 710032, People's Republic of China*
[2] *College of Mechatronics and Automation, National University of Defense Technology, Changsha, Hunan 410073, People's Republic of China*
[3] *Department of Radiology, Xijing Hospital of the Fourth Military Medical University, Xi'an Shaanxi 710032, People's Republic of China*

[†] These authors contributed equally to this work.

* Correspondence to:
Prof. Qing-Rong Tan
Department of Psychiatry, Xijing Hospital, Fourth Military Medical University,
Xi'an, Shaanxi 710032, People's Republic of China
Tel: +86 29 8966 1679
Fax: +86 29 83293951
Email: tanqingr@fmmu.edu.cn
Or
Prof. Dewen Hu,
College of Mechatronics and Automation, National University of Defense Technology,
Changsha, Hunan 410073, People's Republic of China
Tel: +86 731 8457 4992
Fax: +86 731 8457 4992
Email: dwhu@nudt.edu.cn

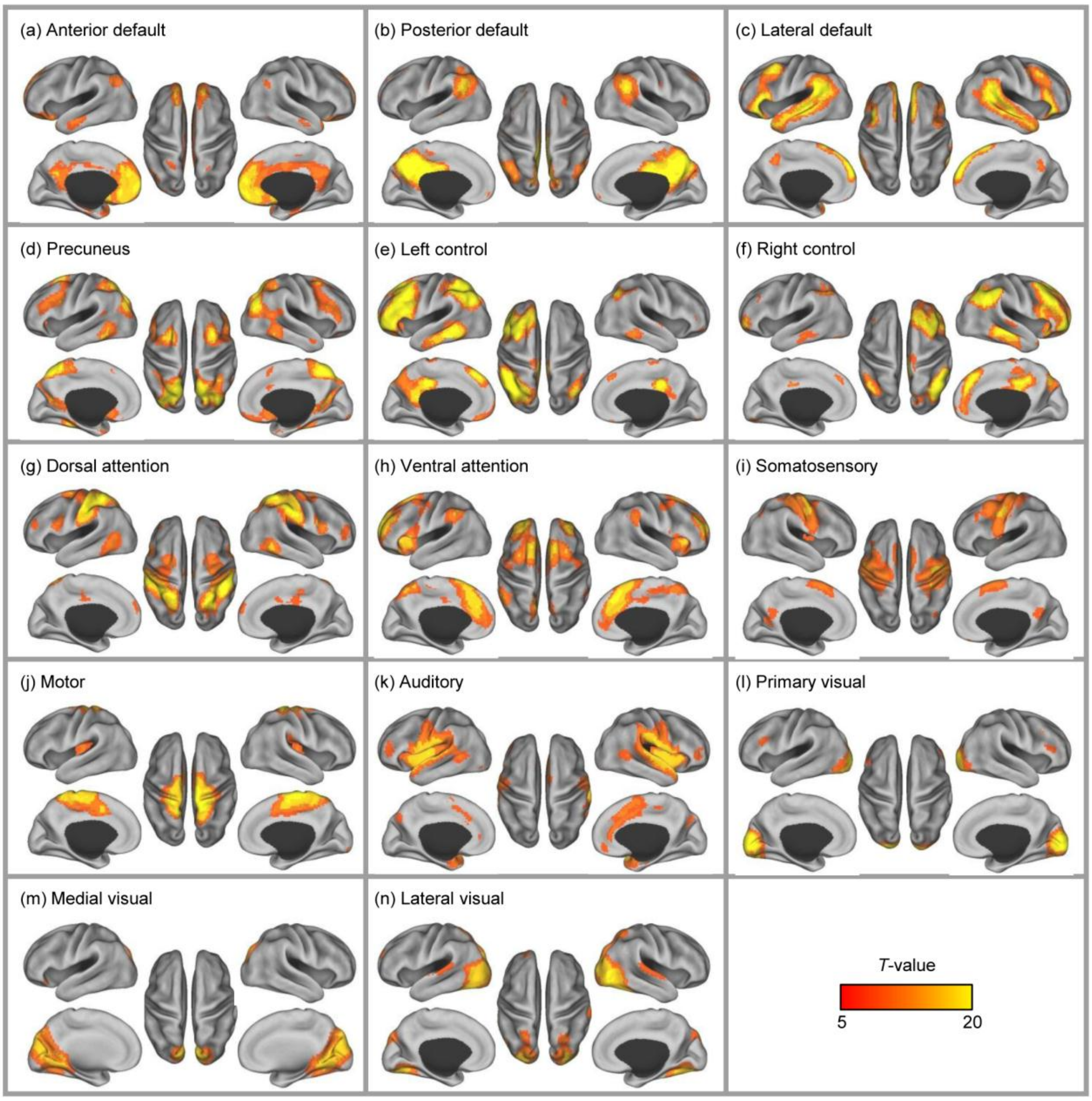

Supplementary Figure 1 Group ICA functionally relevant intrinsic connectivity networks. CARET software (CARET; http://brainvis.wustl.edu) was used for surface rendering.

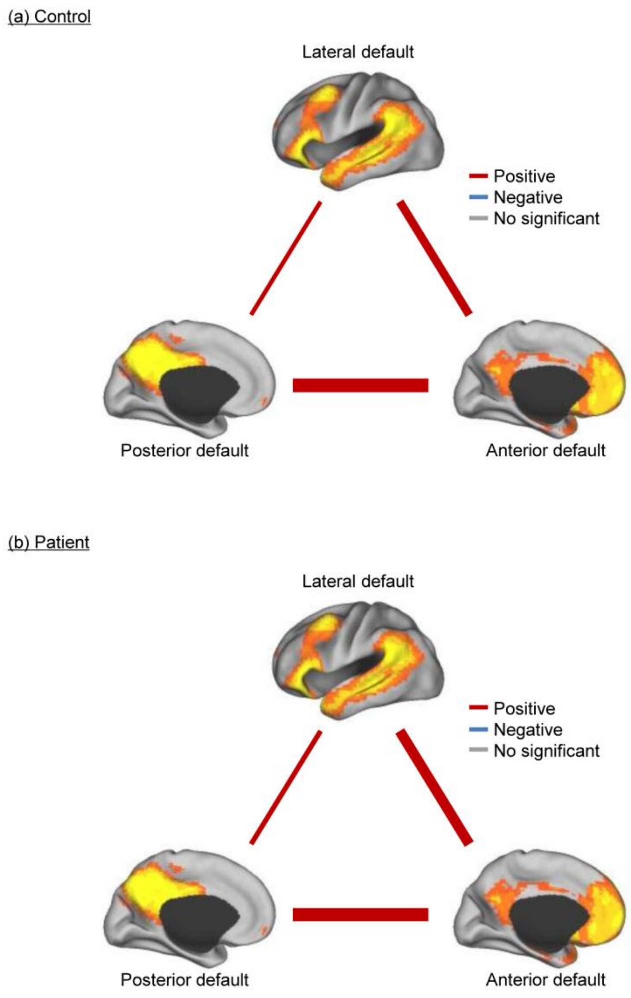

Supplementary Figure 2 Functional connectivity between default mode subnetworks (One-sample *t*-test, $P < 0.05$, FDR corrected; a, control; b, patient). Significant functional connections in the non-medicated patients (n=34) are the same as for all patients. In medicated patients (n=21), the significant functional connections are the same as for all the patients. The red and blue lines represent significant positive and negative correlations, respectively. The gray lines represent that the functional connectivity is not significant. CARET software (CARET; http://brainvis.wustl.edu) was used for surface rendering.

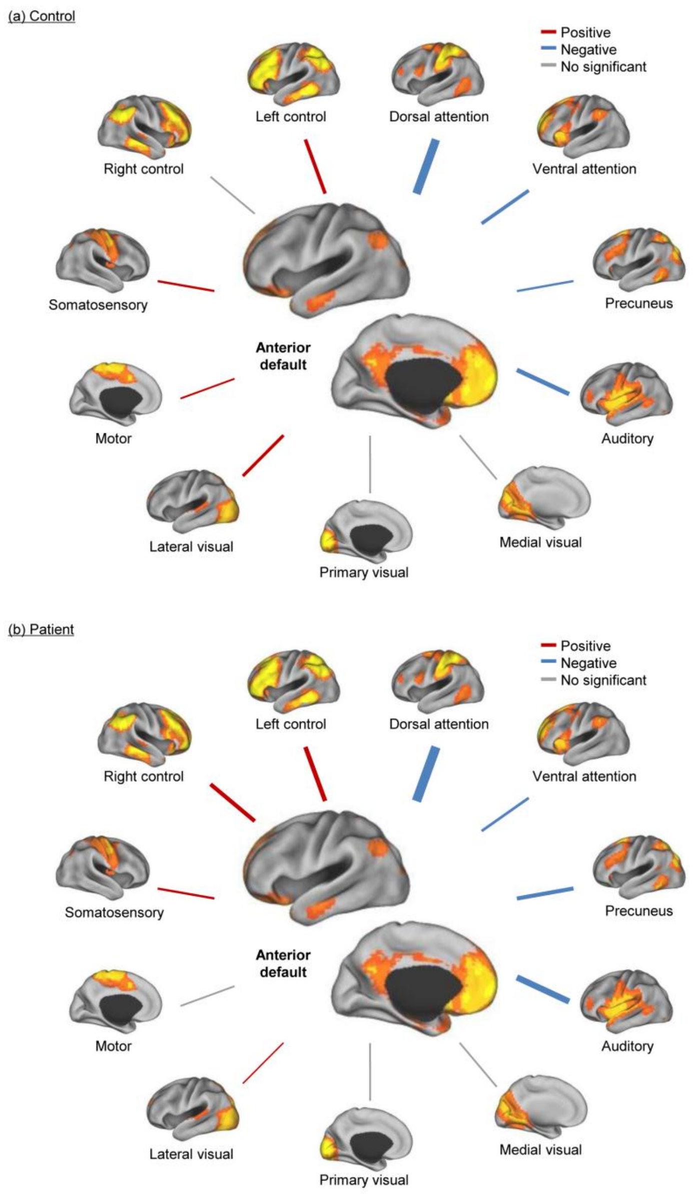

Supplementary Figure 3 Functional connectivity between anterior default mode network (DMN) and other intrinsic connectivity networks (One-sample *t*-test, $P < 0.05$, FDR corrected; a, control, b, patient). Significant functional connections in the non-medicated patients (n=34) are the same as for all patients, with the exception of that between the anterior DMN and ventral attention network. In medicated patients (n=21), the significant functional connections are the same as for all the patients, with the exception of that between the anterior DMN and lateral visual network. The red and blue lines represent significant positive and negative correlations, respectively. The gray lines represent that the functional connectivity is not significant. CARET software (CARET; http://brainvis.wustl.edu) was used for surface rendering.

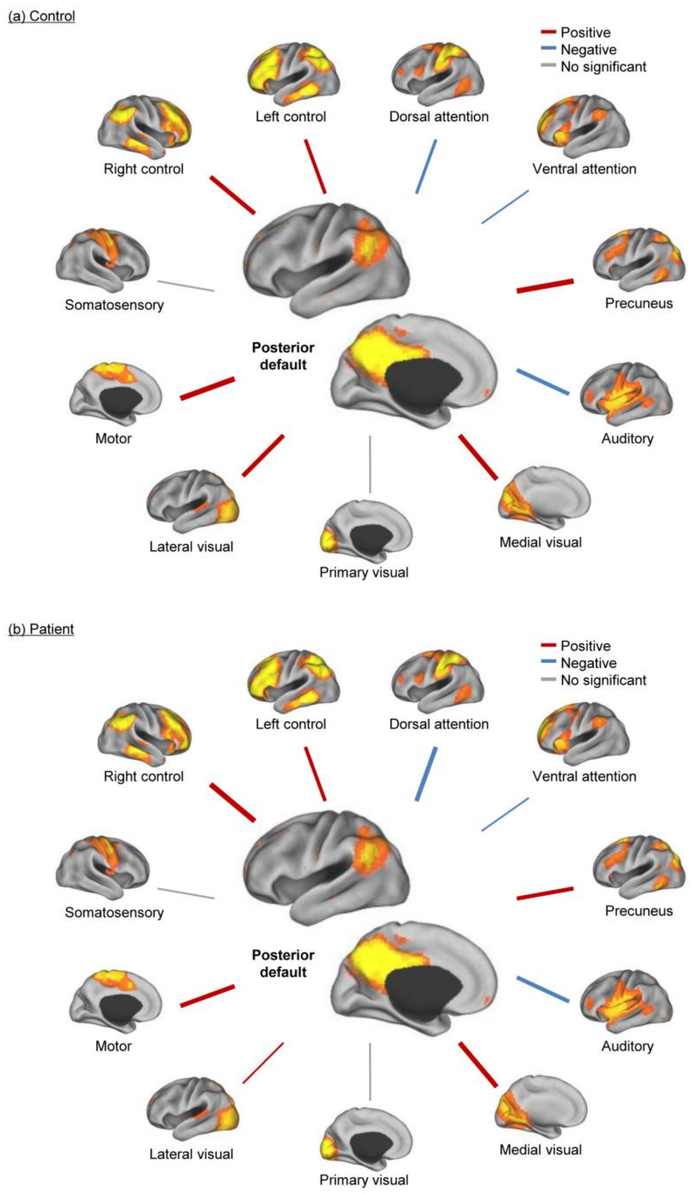

Supplementary Figure 4 Functional connectivity between the posterior default mode network (DMN) and other intrinsic connectivity networks (One-sample *t*-test, $P < 0.05$, FDR corrected; a, control, b, patient). Significant functional connections in the non-medicated patients (n=34) are the same as for all patients, with the exception of that between the posterior DMN and ventral attention network. In medicated patients (n=21), the significant functional connections are the same as for all the patients, with the exception of that between the posterior DMN and lateral visual network. The red and blue lines represent significant positive and negative correlations, respectively. The gray lines represent that the functional connectivity is not significant. CARET software (CARET; http://brainvis.wustl.edu) was used for surface rendering.

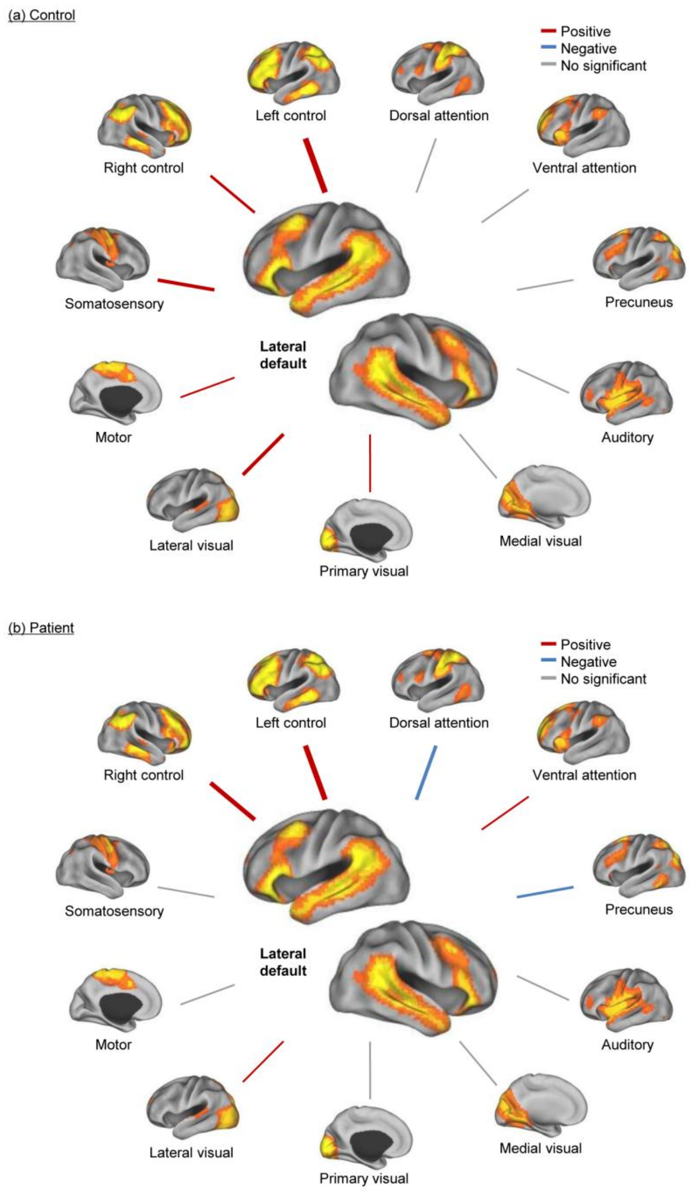

Supplementary Figure 5 Functional connectivity between the lateral default mode network (DMN) and other intrinsic connectivity networks (One-sample *t*-test, $P < 0.05$, FDR corrected; a, control, b, patient). Significant functional connections in the non-medicated patients (n=34) are the same as for all patients, with the exception of that between the lateral DMN and primary visual network (significant additionally). In medicated patients (n=21), the significant functional connections are the same as for all the patients, with the exception of those between the lateral DMN and lateral visual network and between the lateral DMN and ventral attention network. The red and blue lines represent significant positive and negative correlations, respectively. The gray lines represent that the functional connectivity is not significant. CARET software (CARET; http://brainvis.wustl.edu) was used for surface rendering.
